# The integrated stress response in neurodegenerative diseases

**DOI:** 10.1186/s13024-025-00811-6

**Published:** 2025-02-19

**Authors:** Maria Astrid Bravo-Jimenez, Shivangi Sharma, Soheila Karimi-Abdolrezaee

**Affiliations:** Department of Physiology and Pathophysiology, Multiple Sclerosis Research Centre, Rady Faculty of Health Sciences, University of Manitoba, Children Hospital Research Institute of Manitoba, 745 Bannatyne Avenue, Winnipeg, MB R3E 0J9 Canada

## Abstract

The integrated stress response (ISR) is a conserved network in eukaryotic cells that mediates adaptive responses to diverse stressors. The ISR pathway ensures cell survival and homeostasis by regulating protein synthesis in response to internal or external stresses. In recent years, the ISR has emerged as an important regulator of the central nervous system (CNS) development, homeostasis and pathology. Dysregulation of ISR signaling has been linked to several neurodegenerative diseases. Intriguingly, while acute ISR provide neuroprotection through the activation of cell survival mechanisms, prolonged ISR can promote neurodegeneration through protein misfolding, oxidative stress, and mitochondrial dysfunction. Understanding the molecular mechanisms and dynamics of the ISR in neurodegenerative diseases aids in the development of effective therapies. Here, we will provide a timely review on the cellular and molecular mechanisms of the ISR in neurodegenerative diseases. We will highlight the current knowledge on the dual role that ISR plays as a protective or disease worsening pathway and will discuss recent advances on the therapeutic approaches that have been developed to target ISR activity in neurodegenerative diseases.

## Introduction

The integrated stress response (ISR) is an evolutionarily conserved mechanism to cellular stress that is activated by different intrinsic and extrinsic factors. The main function of the ISR is to maintain cellular homeostasis by downregulating protein synthesis and upregulating specific target genes [1]. The key intrinsic stress is endoplasmic reticulum stress (ER stress), which occurs when the capacity of protein folding is exceeded [1, 2]. Extrinsic stressors include glucose and amino acid deprivation, hypoxia, viral infections, and the presence of reactive oxygen species (ROS) [1]. The ISR works with several other cellular adaptation pathways such as proteotoxicity, ubiquitin–proteasome, autophagy, phosphatidylinositol‐3 kinase (PI3K) signaling, and unfolded protein response (UPR); which act in a time-dependent manner upon induction of any stress mediated signaling [3–10]. Moreover, the cross-talk of ISR with osmotic stress response (OSR), DNA damage response (DDR), and heat-shock protein (HSP) response is reported and is often known to function in a cytoprotective manner [11–14].

There are four regulatory ISR-kinases, all of which converge on the phosphorylation of the alpha subunit of the eukaryotic initiator factor 2 (eIF2α) at the serine site 51 [15]. Phosphorylation of eIF2α reduces global protein synthesis and the formation of stress granules are also reported in some cases, while promoting the translation of stress-related genes such as activating transcription factor 4 (ATF4) [16], which supports cell survival. However, under conditions of severe cellular stress, the adaptive response loses its ability to effectively alleviate the stress and instead promotes the activation of apoptotic pathways [17, 18]. The ISR is terminated with the dephosphorylation of eIF2α in a negative feedback loop manner. The ISR has been observed to be activated in different neurodegenerative disorders like multiple sclerosis (MS), Alzheimer’s disease (AD), Parkinson’s disease (PD), and amyotrophic lateral sclerosis (ALS) [19]. This review outlines ISR mechanisms, and its relevance in CNS homeostasis and in neurodegenerative disorders where ER stress and ISR link together to contribute to neuronal cell death, inflammation, protein aggregation, and cognitive impairment. We will also provide an overview on the current therapeutic strategies targeted toward the modulation of dysregulated ISR in neurodegenerative disorders.

### Initiation of the ISR: Four sensors, one phosphorylation

**Fig. 1:**
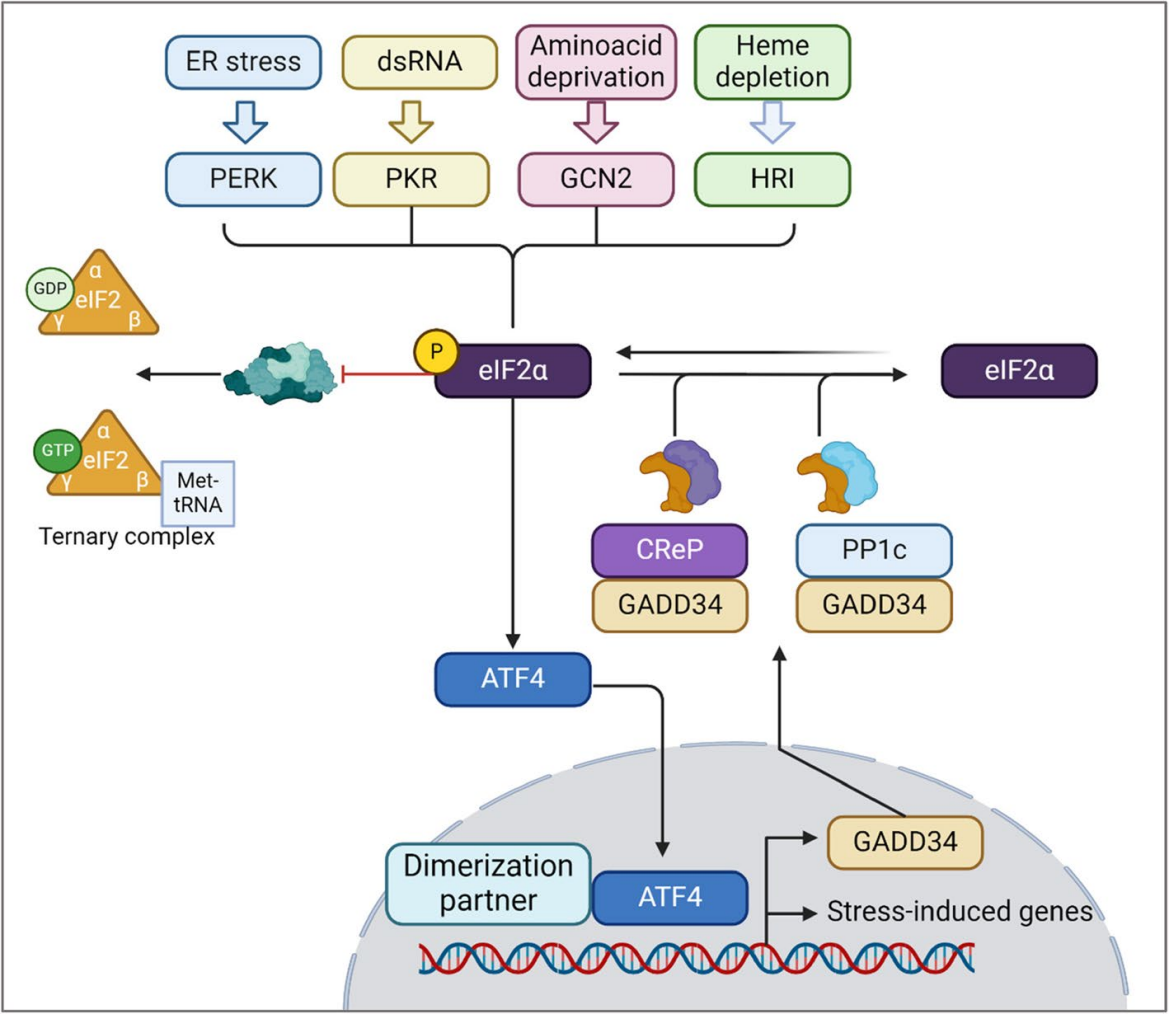
The integrated stress response signaling pathway. The ISR can be initiated upon sensing ER stress, dsRNA, amino acid deprivation, and heme depletion, which are recognized by PERK, PKR, GCN2, and HRI respectively. Once activated, the four kinases converge in the phosphorylation of eIF2α that then activates ATF4. ATF4 translocate to the nucleus in which promotes the translation of stress response genes. Evidence shows regulation of ISR through “negative feedback”. ATF4 induces the expression of GADD34, which promotes the dephosphorylation of eIF2α by recruiting CReP and PP1c, hence restoring protein synthesis. Figure was created in BioRender

The ISR is first initiated by a disturbance in homeostasis and is recognized by the eIF2α protein kinases (EIF2AKs). When changes in homeostasis are detected, the kinases phosphorylate the alpha subunit of the eukaryotic initiator factor 2 (eIF2α) to inhibit protein synthesis, reconfigure gene expression for stress adaptation or inducing apoptosis [1]. The four kinases are PKR-like ER kinase (PERK), protein kinase double-stranded RNA dependent (PKR), general control non-derepressible-2 (GCN2), and heme-regulated inhibitor (HRI) [15, 20]. While each kinase has its own regulatory mechanisms, they all converge on the phosphorylation of eIF2α at the serine site 51 **(**Fig. 1), which is critical for translation control [21, 22]. PERK, also known as EIF2AK3, is an ER transmembrane protein that becomes activated upon detecting disturbances in the ER, like the accumulation of misfolded or unfolded proteins, calcium depletion, or redox imbalance [1, 23, 24]. There are two proposed models for PERK activation. One model suggests that, in homeostatic conditions, PERK remains inactive as its luminal domain is bound to GRP78. Upon ER stress, GRP78 dissociates from PERK to bind with unfolded proteins, leading to PERK dimerization and autophosphorylation that initiates downstream signaling [9, 25, 26]. The second and most recent model suggests an alternative mechanism in which PERK can be activated directly by binding to unfolded or misfolded proteins via its luminal domain [27, 28]. Once activated, PERK then phosphorylates eIF2α (p-eIF2α), preventing the translation of mRNA and reducing protein synthesis, which allows the ER to either refold or dispose of the misfolded proteins [15]. Simultaneously, p-eIF2α initiates the translation of ISR-specific mRNAs, like ATF4 [1].

Unfolded proteins are not the only trigger for PERK autophosphorylation. It has been reported that saturation of lipids and modifications in the lipid composition of the ER membrane can trigger PERK activation [29]. Moreover, early studies reported that glucose deprivation in cultured hippocampal neurons leads to PERK activation and an increased expression of caspase-12, an ER resident caspase that has been associated with stress-induced apoptosis [30]. PKR (EIF2AK2) is found in the cytosol and nucleus of mammalian cells [1, 15, 31]. In addition to mounting an interferon response to viral infection [3], PKR also responds to other stimuli such as metabolic and ER stress [31, 32], as well as oxidative stress [33]. Upon stress, PKR dimerizes and auto-phosphorylates, forming a PKR-PKR complex that phosphorylates eIF2α [15]. Studies have shown that prolonged activation of PKR after oxidative stress increased the sensitivity to apoptosis, making its downregulation important for promoting cell survival [33, 34]. Importantly, PKR induces activation of the pro-apoptotic factor C/EBP homologous protein, CHOP, in response to hyperoxia [35]. GCN2 (EIF2AK4) is proposed to regulate changes in gene expression due to acid and glucose deprivation by sensing uncharged transfer ribonucleic acid (tRNA), although the exact mechanism is yet to be elucidated [15, 36]. Under amino acid deprivation, tRNA accumulates in the A site of the ribosome where it is recognized by the GCN2 regulatory domain histidyl-tRNA synthetase (HisRS), triggering GCN2 dimerization, eIF2α phosphorylation, and ISR activation [4]. GCN2 can also sense other stresses. For instance, inhibiting the proteasome system leads to the formation of stress granules that are primarily recognized by GCN2 and initiates the ISR [37, 38]. HRI or EIF2AK1 is mainly found in erythroid cells. Early studies reported that HRI is involved in erythrocyte differentiation and is required to produce α and β globin in red blood cell (RBC) precursors and it also promotes the survival of these cells under heme deficiency [39]. When the availability of iron decreases, eIF2α phosphorylation by HRI inhibits the translation of globin mRNAs, prevents hemoglobin production and exerts protection against toxic globin aggregates [1, 39]. HRI has also been found to initiate autophagy in cases when α-synuclein is overexpressed, hence helping to clear out the protein aggregates accumulated in the cytosol [40]. It is also demonstrated that HRI is required for inflammatory responses during infection [41]. Recent studies show that HRI acts as a proteotoxicity sensor via a pathway involving Hsp70, Bag3 and HRI, which detects the abnormal accumulation of proteins in the cytosol and triggers the phosphorylation of eIF2α [42]. All four kinases phosphorylate eIF2α at its serine site 51 and with this in common, they can often overlap and act cooperatively to sense different stress stimuli and integrate them to achieve specific cell responses, hence the name “integrated stress response”.

### Downstream p-eIF2a, cellular responses, and termination of the ISR

Under homeostatic conditions, eIF2 participates in mRNA translation and recognition of the initiation codon AUG [43]. eIF2 is a 126 kDa heterotrimer protein comprised of α, β and γ subunits [44] with the eIF2α subunit playing a major regulatory role due to its RNA binding and phosphorylation sites [1]. Translation initiation involves the assembling of elongation-competent 80S ribosomes with an initiator tRNA at the ribosomal P site 1. This two-step process requires at least 9 eukaryotic initiation factors including eIF2. The first step requires the formation of 48S initiation complexes, which then join 60S subunits [43]. Many of the 48S initiation complexes are formed by a 43S preinitiation complex which is comprised of a 40S subunit, the eIF2-GTP-Met-tRNA_i_^Met^ ternary complex, and other eIFs like eIF3, eIF1, eIF1A, and eIF5. This complex is composed of eIF2, guanosine triphosphate (GTP), and charged methionyl-transfer RNA (Met-tRNA_i_^Met^) [43, 45]. Upon stress, p-eIF2α alters the regular translation initiation by inhibiting the formation of active GTP from the eIF2-GDP complex and binds strongly to a modulatory portion of eIF2β that inhibits the formation of active GTP from the eIF2-GDP bound form. Consequently, there is less availability of the ternary complex, which leads to decreased translation rates (Fig. 1**)** and increased translation of ISR-related mRNAs like ATF4, ATF5, CHOP, and GADD34 [1, 45].

**Fig. 2:**
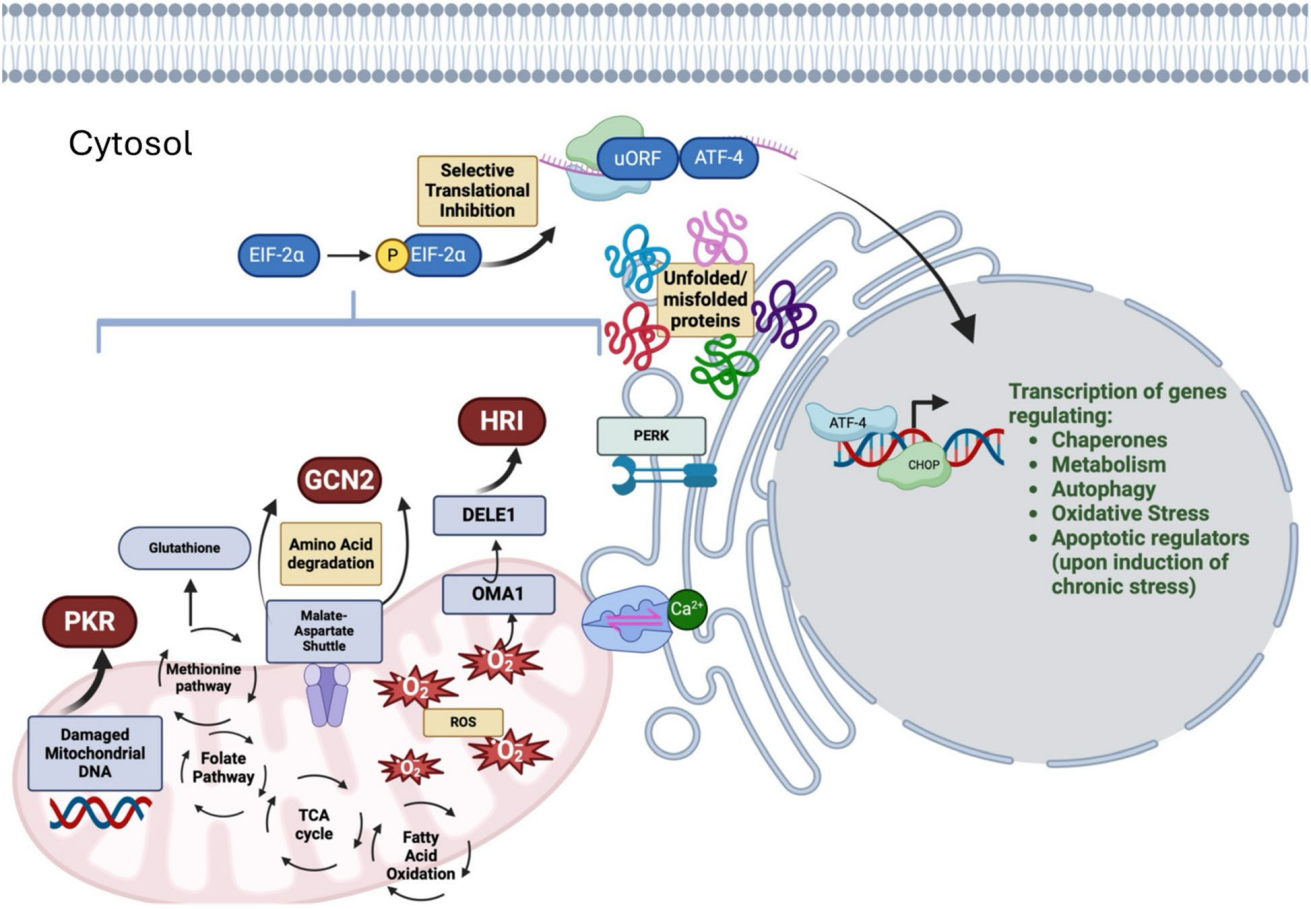
Mitochondrial dysfunction and ISR: The activation of the integrated stress response (ISR) during mitochondrial dysfunction is triggered by various mechanisms. The fragmentation of mitochondrial DNA is among the leading causes of mitochondrial dysfunction, which is managed by an ISR sensor, protein kinase RNA-activated (PKR). Mitochondrial dysfunction during amino acid metabolism is managed by another class of ISR kinase- general control nonderepressible-2 (GCN2). The Tri-Carboxylic Acid (TCA) cycle is fed via the amino acid degradation during metabolic rewiring stage of mitochondrial stress, and the depletion of amino acids results in activation of GCN2. During this process, the equilibrium maintenance of reducing equivalents is maintained by the malate and aspartate shuttle. Upon induction of mitochondrial stress by generation of reactive oxygen species (ROS), a mitochondrial protease known as OMA1 regulates the mitochondrial stress dynamics by cleaving DAP3-binding cell death enhancer 1 (DELE1). This subsequently activates heme-regulated inhibitor (HRI) after translocating to the cytoplasm. The crosstalk between mitochondria and endoplasmic reticulum aids ER to sense the alterations in the levels of calcium (Ca2 +), ROS, and changes in energy productions; leading to activation of ER transmembrane protein- PKR-like ER kinase (PERK). Upon mitochondrial dysfunction and the ER-mitochondrial crosstalk, these protein kinases phosphorylate eIF2*α* and inhibit global protein translation to mitigate stress and restore the normal homeostasis. Until the stress is resolved, a translational shift due to upstream open reading frame (uORF) mediates selective translation of proteins such as ATF-4 and usurps the global translation to mitigate the stress. ATF-4 along with its other dimerization partners regulates amino acid synthesis genes, antioxidant pathways, chaperones and metabolism related genes to restore the homeostasis post stress. However, chronic stress results in activation of CHOP-mediated apoptotic cell death. Figure was created in BioRender

ATF4 is a key regulator in the ISR network, as it is vital for relieving ER stress by either promoting adaptation or triggering apoptosis [1]. ATF4 downstream activity initiates with the formation of homo and or heterodimers with other basic leucine zipper (bZIP) transcription factors including CHOP or AP-1 members that are known to regulate the transcriptional selectivity and thereby influence the outcome of ISR [46, 47]. The interacting heterodimers bind to cAMP (cyclic adenosine monophosphate) responsive elements to control target gene expression [18], which leads to the transcriptional upregulation of stress-related genes and pathways related to amino acid transport and metabolism as ATF4 can promote cell survival by inducing autophagy. Following amino acid starvation and ER stress, GCN2 and PERK along with ATF4 and CHOP can promote the expression of genes related to the autophagosome formation and function [48]. Dimer combinations between ATF4 and CHOP can regulate transcription through various mechanisms. In response to leucine starvation, ATF4-CHOP heterodimers regulate genes related to the degradation of ubiquitinated substrates, such as Nbr1, Atg7, and p62 [48]. Autophagy-related genes Atg10, Gabarap, and Atg5 are expressed after the formation of ATF4-CHOP heterodimer in response to amino acid deprivation [48]. Other reports suggest that ATF4 alone can target genes related to amino acid transport and biosynthesis, and it can act along with CHOP to regulate several shared genes related to protein synthesis, mRNA translation, and the unfolded protein response (UPR) [17]. The UPR is a signaling pathway that becomes activated due to ER stress and detects unfolded proteins through ER transmembrane receptors: PERK, IRE1, and ATF6. Both the ISR and UPR converge in the phosphorylation of eIF2α through PERK (Fig. 2) [1]. eIF2α phosphorylation can either promote cell survival or cell death. The increase in protein synthesis mediated by ATF4 and CHOP can promote cell death by oxidative stress and depletion of ATP [1, 18, 49]. This dual role of ATF4 has been attributed to the formation of heterodimers with different binding partners that lead to different responses. Binding partners of ATF4, such as C/EBPβ and C/EBPγ are involved in signal adaptation, upregulation of stress response genes, and protection against oxidation [50, 51]; while ATF4-CHOP is mainly associated with autophagy and pro-apoptotic activity. As reviewed previously by others [52], CHOP has been identified to induce apoptosis; however, as described earlier, it can also promote cell survival. This discrepancy may reflect the duration and level of stress, as well as the level of CHOP expression. These findings suggest that ATF4/CHOP may promote an initial survival response; however, with prolonged stress CHOP expression will initiate cell death to restore homeostasis [52].

The termination of the ISR, restoration of protein synthesis, and return to homeostasis occur upon eIF2α dephosphorylation in a process regulated by the protein phosphatase 1 (PP1) complex through its catalytic subunit (PP1c). The phosphatase activity can be regulated by GADD34 (growth arrest and DNA damage-inducible protein 34) or by the “constitutive repressor of eIF2α phosphorylation”, known as CReP [53, 54]. CReP forms a complex with PP1c to enable homeostasis by maintaining low activity of p-eIF2α, whereas GADD34 expression is induced during the late stages of the ISR in response to ATF4 and forms a complex with PP1 to promote eIF2α dephosphorylation. Thus, this negative feedback loop appears to be essential for restoring homeostasis after a stress response [1, 53].

### ISR and mitochondrial UPR

Mitochondrial stress responses (MSRs) can also result in the buildup of misfolded or damaged proteins. Mutations or deletions in mitochondrial DNA (mtDNA) causes an accumulation of unfolded proteins, which can trigger the activation of the mitochondrial unfolded protein response (UPR^mt^) [55]. Growing evidence has implicated ISR in UPR^mt^ [56, 57] (Fig. 2). Studies in *S. cerevisiae* and *C. elegans* indicate that UPR^mt^ is regulated by ISR sensors or eIF2α kinases. GCN2 depletion is known to significantly upregulate the expression of mitochondrial chaperones that activates UPR^mt^ [55]. Another study revealed that during mitochondrial stress, activation of HRI, even with the absence of full heme, attenuates the global protein translation through phosphorylation of eIF2α, thus implicating a cross-talk between ISR and mitochondrial dysfunction [58]. During the active state of ISR and upon diverse mitochondrial insults, the upstream open reading frames (uORF’s) are involved in translation of selective transcription factors such as ATF4, ATF5 and CHOP [59–61]. The accumulation of misfolded or unfolded proteins in the mammalian mitochondrial matrix often leads to the upregulation of chaperones in mitochondria but no ER stress protein response is initiated [61]. Interestingly, UPR^mt^ activation also leads to a reduction in the transcription of oxidative phosphorylation (OXPHOS) components, as mitochondria attempt to lessen their functional demands while addressing the stress [62, 63]. The mitochondrial stress modulators such as FCCP, doxycycline, MitoBloCk and actinonin are known to activate ATF4 and suppress the mitochondrial translation, alleviating the burden of misfolded proteins [62, 64]. ATF5 is also known to have direct effects on UPR^mt^, as inhibiting its function suppresses the induction of UPR^mt^-related genes during the mitochondrial stress. However, ATF5 overexpression in *C. elegans* lacking ATFS-1 is reported to restore the expression of HSP60 [62, 64]. These findings indicate a dual functionality of ATF-5 (or its isoform ATFS-1 in *C. elegans)* in regulation of mitochondrial dysfunction that needs further examination. Altogether, mitochondrial dysfunction and its association with other organelles that initiate stress response cascade have been reported in various disease conditions including neurodegenerative disorders [65–67], which is further discussed in detail in neurodegenerative disease section.

### Pharmacological modulation of the ISR

The ISR has been associated with inflammation, cancer, diabetes, and neurodegenerative disorders. Hence, the ISR has become a promising therapeutic target [45]. Due to the dual roles that the ISR plays in promoting cell survival or cell death, different strategies have been developed to either enhance or suppress the ISR by targeting eIF2α, eIF2α kinases, or ATF4 [1] (Table 1). Pharmacological enhancement of the ISR can be attained by direct targeting of eIF2α kinases or by prolonging the phosphorylation of eIF2α with phosphatase inhibitors [1]. A common ISR activator is CCT020312, a small molecule that selectively targets PERK and promotes the phosphorylation of eIF2α without eliciting a general UPR [68]. BTdCPU is an active N,N’-diarylurea that acts as a potent HRI activator [69]. Drugs like salubrinal, guanabenz, and Sephin1 are known to prolong the ISR, which is achieved by inhibiting eIF2α dephosphorylation [1]. Salubrinal is a phosphatase inhibitor that selectively hinders eIF2alpha dephosphorylation independent of the eIF2α kinases. Although its mechanism of action is yet to be elucidated, evidence suggests it seems to inhibit CReP-PP1 and GADD34-PP1 complexes [70]. Guanabenz, a drug initially used as a treatment for hypertension, is shown to increase eIF2α phosphorylation by inhibiting GADD34 [71]. However, Guanabenz does not selectively inhibit GADD34 and its affinity for the α2-adrenergic receptor can result in adverse effects like drowsiness and lethargy upon overdose [72]. Hence, Guanabenz derivatives have been investigated. Sephin1 is a promising Guanabenz derivative that selectively inactivates the binding of GADD34 to PP1c without causing α2-adrenergic-related side effects in vitro or in vivo [73]. Taken together, Salubrinal, Guanabenz, and Sephin1 prolong the ISR and appear to decrease protein synthesis and allow protein folding, thus they are currently the most promising candidates to mitigate proteotoxicity [1].

**Table 1:** Pharmacological modulation of the ISR

Compound	Target	Mechanism	Reference
Enhancer			
CCT020312	PERK	Promotes eIF2α phosphorylation without eliciting a general UPR	Stockwell, S., et.al. [68]
BTdCPU	HRI	Induces eIF2α phosphorylation by activating HRI (as observed in free cell lysates)	Chen, T. et al., 2011 [69]
Salubrinal	GADD34-PP1 and CreP-PP1	Prolongs the ISR by inhibiting GADD34-PP1 and CReP-PP1 complexes, which in turn inhibits the dephosphorylation of eIF2α	Boyce, M et.al., 2005 [70]
Guanabenz	GADD34	Prolongs the ISR by the non-selective inhibition of GADD34, which leads to the dephosphorylation of eIF2α	Tsaytler, P., et.al., 2011 [71]
Sephin1	GADD34	Prolongs the ISR by the selective inhibition of GADD34, which leads to the dephosphorylation of eIF2α	Das, I., et.al., 2015 [73]
Inhibitor			
ISRIB	eIF2Bδ	Acts as an allosteric antagonist of peIF2α by targeting the eIF2B complex	Sidrauski, C. et al., 2013 [74] Sekine, Y., 2015 [75], Tsai, J.C, 2018 [76], Zyryanova, A.F., 2018 and 2021 [77, 78] & Rabouw, H. et al., 2019 [79]
GSK2606414	PERK	ATP-competitive PERK inhibitors prevent PERK autophosphorylation and hence inhibiting the phosphorylation of eIF2α	Axten, J. M., et.al, 2012 [80] & 2013 [81]
C16	PKR	Inhibits the ISR by blocking the autophosphorylation of PKR	Jammi, N. V, et.al, 2003 [82]
A92	GCN2	Can selectively silence GCN2 but also induces ISR by activating PERK	Szaruga, M., et.al., 2023 [83]

While the ISR can have cytoprotective effects, its chronic activation can result in neurological disorders. Hence, pharmacological inhibitors of the eIF2α kinases have pursued as therapeutic targets [1]. One of the most well-known ISR inhibitors is the integrated stress response inhibitor (ISRIB). First identified by Sidrauski and colleagues, ISRIB acts downstream of all ISR-kinases and reverses the outcomes of eIF2α phosphorylation and restoring translation [74]. Several studies have demonstrated that ISRIB binds to the eIF2B complex, a guanine nucleotide exchange factor (GEF) for eIF2, and it stabilizes it in a structure that can exchange GDP for GTP on eIF2, thus promoting the restoration of protein synthesis [75–78]. ISRIB has been demonstrated to inhibit the formation of stress granules due to eIF2α phosphorylation and is considered a promising candidate treatment for diseases associated with the formation of protein aggregates [74]. However, recent studies have shown that ISRIB can only suppress the ISR when the levels of p-eIF2α are below a critical threshold [79]. Additionally, by using an ATF4 reporter cell line, it was found that ISRIB could not hinder the expression of stress-induced proteins under high intracellular levels of p-eIF2α. Thus, ISRIB seems to be effective under conditions of limited stress, and further investigation is required to determine if this property would affect its efficacy when used as a treatment for ISR-related diseases [79]. Other eIF2α inhibitors include the ATP-competitive PERK inhibitor GSK2606414 which prevents PERK autophosphorylation [80, 81] or the small molecule C16 which blocks autophosphorylation of PKR [82]. A recent work has reported that the commonly used ATP-competitive PERK inhibitorGSK2606414, can effectively inhibit PERK when used in nanomolar concentrations; however, it could activate GCN2 when used in micromolar concentrations [83]. Similarly, the PKR inhibitor, C16, was found to activate GCN2. In contrast, the GCN2 inhibitor A92 was shown to be effective in silencing its target but ultimately induces the ISR by activating PERK [83]. Altogether, extensive progress has been made in modulating the ISR in different preclinical in vivo and in vitro models **(**Table 1**)**. Nonetheless, the complexity and redundancy of the stress-sensing pathways of the ISR requires further investigation in preclinical studies to help refine the selectivity of activators and inhibitors in future clinical settings.

### The ISR in CNS homeostasis

The ISR pathway is fundamental for maintaining CNS homeostasis during neurodevelopment and adulthood and play important roles in CNS pathology [84]. Developmental studies suggest the existence of a non-canonical eIF2α pathway that selectively modulates changes in protein synthesis in axons and is required for neural wiring [85]. As an example, Sempahorin-3A (Sema3A), an extrinsic cue that can initiate protein synthesis in axons, is shown to induce eIF2α phosphorylation by activating PERK [85]. However, activation of sema3A signaling does not result in the suppression of global protein translation, nor the translation of ATF4. ISR is also implicated in the onset of some neurodevelopmental disorders such as Fragile X syndrome (FXS) and Down’s syndrome [86]. These disease models are known to exhibit impaired synaptic plasticity, which are hypothesized to be central to the clinical symptomatology [86].

Homeostasis of the CNS and proper function of neural network rely on synaptic transport and communication, which itself requires turnover of large amounts of proteins [87]. Evidence shows that the ISR can be activated by an abnormal increase in protein synthesis [84]. Given memory formation requires continuous protein synthesis there has been a growing interest in unraveling whether ISR plays a role in memory. Current findings suggest that the ISR negatively regulates memory formation. Recent research underscores the critical role of translation, particularly the bidirectional function of eIF2α phosphorylation, in long-term memory potentiation (LTP) and synaptic plasticity [88]. Synaptic plasticity is an underlying process of experience-dependent synaptic strength remodeling that is crucial for learning and memory processes. A nuanced interaction of released neurotransmitters, variety and their number of postsynaptic receptors could potentially cause the overall consolidation or diminishment of synaptic connections [89, 90]. *De-novo* protein synthesis is often required for long lasting synaptic plasticity through long-term potentiation (LTP; strengthening of synapse) and long-term depression (LTD; weakening of synapse) [91]. The ISR is known to regulate these protein synthesis pathways, and its effects have been extensively studied in a variety of brain regions of a broad range of species of organisms [19, 92–94].

Studies in the mouse forebrain demonstrate that the specific inhibition of C/EBP-family proteins facilitate long-term plasticity and memory by decreasing ATF4 expression, enhancing hippocampal-dependent spatial memory, and decreasing LTPs threshold [95] GCN2 also contributes to regulating synaptic plasticity, learning, and memory in mice through the modulation of the ATF4/CREB pathway [87]. In contrast, a reduction in the phosphorylation of GCN2 and eIF2α is associated with an LTP-induced stimulus, which can enhance both synaptic plasticity and memory formation [87]. Short-term working memory is a temporary process that does not rely on protein synthesis [96]. It is, however, highly dependent on Ca2 + dynamics. Interestingly, ablation of PERK from the forebrain of adult mice impairs working memory. Although the underlying mechanisms are yet to be identified, it is speculated that PERK mediates working memory by modifying the Ca2 + dynamics [96]. ATF4 is also involved in neurodevelopment by regulating the post-synaptic development of dendritic spines that regulate neuronal activity and are important for synaptic plasticity and memory [97, 98]. Injection of shRNA targeting PERK into the CA1 region of the hippocampus of young adult mice is shown to decrease PERK expression in excitatory and inhibitory neurons, which results in enhanced neuronal excitability and improved cognitive function and hippocampal-dependent learning [99].

Astrocytic protein synthesis plays a major role in modulation of synaptic plasticity and long-term memory consolidation [35]. In a mouse model of genetically ablated Ser51 phosphorylation site of eIF2α (*Eif2a*^*A/A*^) in astrocytes, a reduced p-eIF2α expression resulted in increased protein synthesis which accounts for increased excitatory and decreased inhibitory synaptic outputs to the pyramidal neurons [100]. This highlights the role of autonomous and cell-type specific translational control in memory consolidation [35, 100, 101]. In addition to memory consolidation, astrocytes play a crucial role in maintaining CNS homeostasis by various mechanisms including metabolic support of neurons and maintaining the integrity of the blood brain barrier (BBB) [102]. Upon specific stimuli, including the activation of the ISR, astrocytes become reactive. Astrocyte reactivity in neurodegenerative diseases is often associated with disruption of BBB that promotes neuroinflammation and neurodegeneration [102]. Modulators of the ISR are shown to have cytoprotective effects in CNS injuries and diseases by restoring BBB. For example, studies in a mouse model of CNS stab injury showed that Salubrinal can restore the BBB by increasing the expression of fibronectin and reducing the activation of microglia and macrophages [103]. Likewise, in a rat model of cerebral ischemia, administration of Salubrinal attenuated the expression levels of metalloprotease 9 (MMP-9); a known marker of BBB impairment, as well as adhesion molecules and mediators of leukocyte migration factors, namely ICAM-1 and VCAM-1 [104]. These findings suggest a potential for Salubrinal, as an ISR modulator, in restoring BBB [104–107].

ATF-4 is also known to play an evolutionary conserved role in maintaining CNS metabolism and cellular redox capacity by regulating amino acid biosynthesis, cysteine in particular, with antioxidant effects [108, 109]. In *Parkin* and *Pink* mutants of Parkinsonian *Drosophila* models with a dysfunctional mitochondria-mediated oxidative stress, ATF4 rescues the loss of dopaminergic neurons [108, 109]. In a model of amino acid deprivation, GCN2 also supports amino acid metabolism by its direct interaction with deacetylated tRNAs through histidyl tRNA synthetase (HisRS-like) [110, 111]. A growing body of evidence from animal models of neurodegenerative diseases indicates that sustained phosphorylation of eIF2α is linked to cognitive deficits [112, 113]. The protective effects of GCN2-mediated ISR signaling in nmf205 mouse model of neurodegeneration offers a unique perspective on how the interaction of ribosome with GCN2 acts as a feedback loop during ribosome stalling mRNA translation [114, 115]. In a prion disease mouse model, GADD34 overexpression alleviates neuronal loss and translation and synaptic impairments [116]. Taken together, emerging research has established a role for ISR in CNS development and homeostasis. Recent findings have also uncovered an important role for ISR in CNS pathologies including neurodegenerative diseases [114, 115].

### The role of the ISR in neurodegenerative disorders

**Fig. 3:**
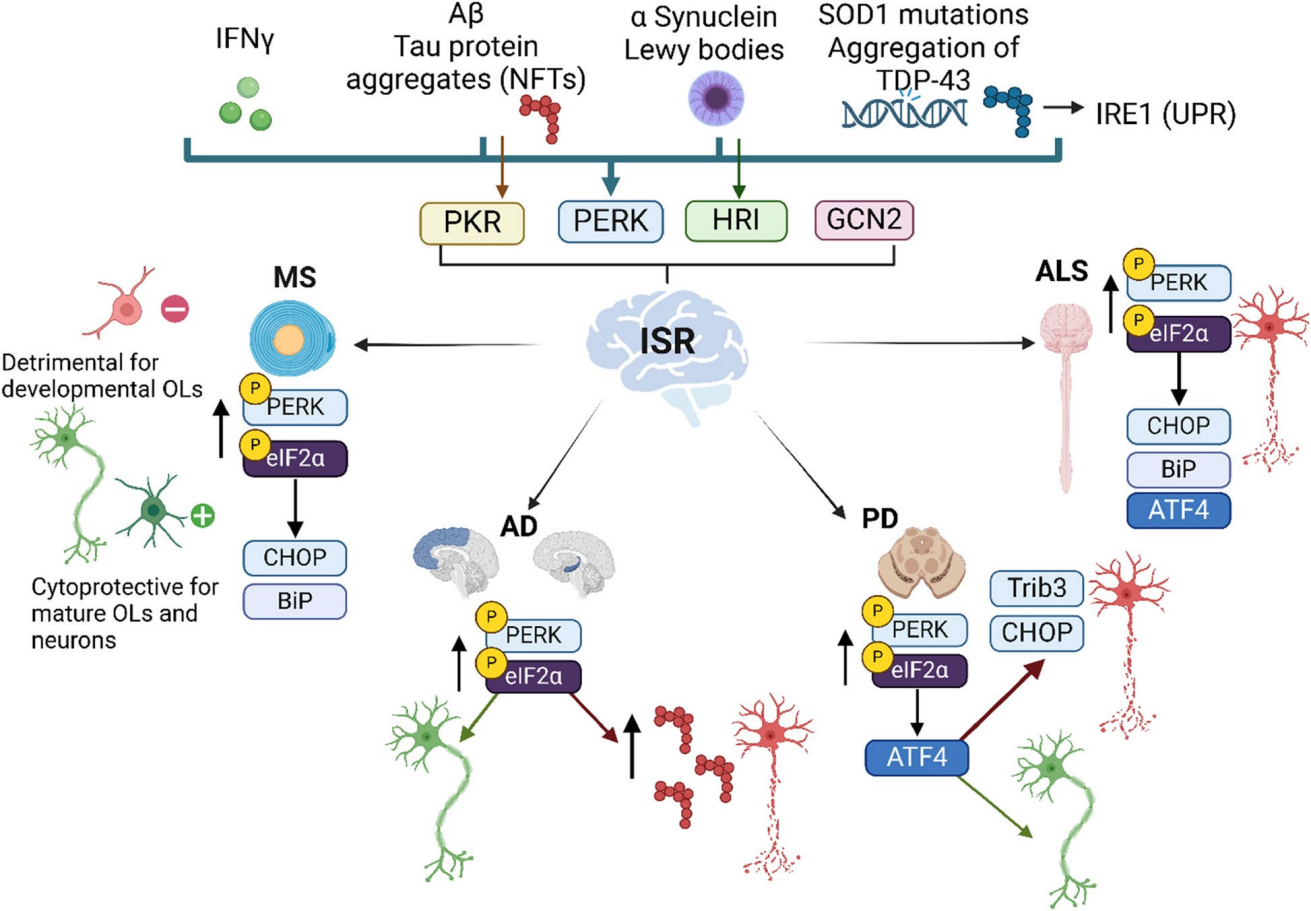
The integrated stress response in neurodegenerative disorders. The integrated stress response (ISR) can be initiated upon sensing ER stress. In Multiple Sclerosis (MS), IFNγ is shown to induce ER stress in myelinating oligodendrocytes (OLs) via the phosphorylation of PERK and eIF2α. The main downstream markers identified in lesions of human MS and the MS animal model EAE (experimental autoimmune encephalomyelitis) are CHOP and BiP. EAE studies show that the ISR can be either detrimental for developing oligodendrocytes or cytoprotective for neurons and mature oligodendrocytes. In Alzheimer’s Disease (AD), the formation of amyloid beta (Aβ) plaques and Tau protein aggregates can initiate ISR primarily through PERK and PKR. ISR markers such as p-PERK and p-eIF2α have been found mainly in the frontal cortex and hippocampus. Evidence implicates PERK activation as either neuroprotective by mitigating Aβ- and tau-induced neuronal death (neuron in green) or cytotoxic by promoting tau phosphorylation and aggregation that lead to neuronal damage (neuron in red). In Parkinson’s Disease (PD), α synuclein and Lewy bodies promote the initiation of the ISR via PERK and HRI phosphorylation. ISR markers are commonly found in the affected substantia nigra and dopaminergic neurons. The PERK-ATF4 signaling pathway is relevant in PD. The involvement of ATF4 in the transcription of Parkin (a pro-survival protein) indicates a cytoprotective role (neuron in green). However, excessive ATF4 activity may contribute to neuronal death by promoting the transcription of pro-apoptotic factors such as Trib3 and CHOP (neuron in red). In Amyotrophic Lateral Sclerosis (ALS) motor neurons in the spinal cord are primarily affected. The ISR is activated by protein aggregation, induced mainly by mutations in TDP-43, SOD1, and FUS. The main markers observed in ALS models and human ALS samples are p-eIF2α, ATF4, CHOP, and BiP. The ISR is mostly detrimental in ALS, and the activation of CHOP in ALS models is linked to apoptosis and neurodegeneration (neuron in red). Figure was created in BioRender

Dysregulation of ISR signaling, particularly eIF2α phosphorylation, has been linked to many neurological disorders that are characterized by neuroinflammation, disturbances in protein homeostasis and oxidative stress including multiple sclerosis (MS), Alzheimer's disease (AD), Parkinson’s disease (PD), amyotrophic lateral sclerosis (ALS), Huntington disease, vanishing white matter disorder, frontotemporal dementia, neurotrauma, and prion disorders [19, 45]. In neurodegenerative conditions, phosphorylation of eIF2α is a common feature in the canonical adaptive signaling cascade of ISR. Chronic activation of eIF2α contributes to the phenotypic characteristics of neurodegeneration such as neuronal cell death and impaired memory [19]. Downstream signaling of phospho-eIF2α-mediated translation inhibition promotes accumulation of proteins, stress granules, liquid droplets, amyloidogenic processing and neuroinflammation that are associated with neurodegenerative disorders [1, 19, 117–119]. Here, we will discuss recent findings on the role and therapeutic potential of ISR in MS, AD, PD and ALS **(**Fig. 3**)**.

### Multiple sclerosis (MS)

MS is an immune-mediated demyelinating disorder of the CNS that results in progressive neurodegeneration [120]. The pathophysiology of MS is characterized by the peripheral activation and infiltration of leucocytes into the CNS, which triggers an inflammatory response and induces the secretion of proinflammatory cytokines that leads to demyelination, oligodendrocyte death, and neurodegeneration [121]. Early reports showed that the ISR-related markers BiP (the expression of which occurs in a constitutive manner), and CHOP (a marker of ER stress) are expressed in post-mortem MS lesions, with a significant increase of CHOP expression in the edges of chronic active lesions [122]. Follow-up studies in the MS animal model, experimental autoimmune encephalomyelitis (EAE) confirmed an increase in the expression of p-eIF2α in EAE lesions [123]. These studies also showed significantly higher expression of CHOP and p-eIF2α in spinal cord neurons of EAE mice.

IFNγ is a key proinflammatory cytokine that drives the pathogenesis of MS and EAE and contributes to demyelination. IFNγ is shown to induce ER stress and promotes apoptosis in developmental rat oligodendrocytes in vitro [124]. Additionally, embryonic mice that received IFNγ ectopically showed hypomyelination, increased expression of the ER-related markers p-eIF2α, BiP, and CHOP, and the apoptosis-related marker Caspase 12 at postnatal day 14 [124]. This evidence indicates a link between hypomyelination, ER stress and IFNγ-induced apoptosis in developmental oligodendrocytes. Further studies in the CNS of adult mice also showed IFNγ induces a moderate ER stress, which activates the ISR via PERK–p-eIF2α ISR in mature oligodendrocytes [125]. However, in this case, the IFNγ-mediated ISR response did not decrease oligodendrocyte survival during EAE. In fact, the activation of the ISR turned out to be protective against EAE-induced demyelination and axonal damage [125]. These two studies show the striking dichotomy in the role of IFNγ-mediated activation of the ISR. While the activation of this pathway is detrimental for developmental oligodendrocytes, it protects mature oligodendrocytes during EAE. This contrasting effect suggests that the IFNγ-mediated ER stress response and the ISR in oligodendrocytes is context-dependent, being detrimental during development but potentially neuroprotective in the context of chronic inflammatory diseases, such as MS.

Interestingly, GADD34-meidtaed dephosphorylation of p-eIF2α is selectively upregulated in myelinating oligodendrocytes of mice that ectopically express IFNγ in the CNS. GADD34-deficient mice at postnatal day 21 also show higher expression of p-eIF2α in oligodendrocytes in the presence of IFNγ, which is associated with reduced oligodendrocyte loss and hypomyelination [126]. These findings demonstrate that enhancing the ISR could promote oligodendrocyte survival in the presence of an MS-relevant cytokine [126]. On the contrary, genetic inactivation of PERK in oligodendrocytes worsens the EAE disease course as 6-week-old PERK-deficient mice show more severe clinical scores and earlier EAE onset with exacerbated demyelination and axonal degeneration compared to their controls [127]. Considering the benefit of ISR activity in oligodendrocytes, further investigation has been conducted to identify the effects of prolonged ISR. Interestingly, these studies show that sustained ISR by prolonging eIF2α phosphorylation can delay the onset of clinical symptoms in 7-week-old EAE mice, reduce oligodendrocyte and axonal loss, and decrease the numbers of T cells in the CNS [128]. More recent findings show that prolonging the ISR can also enhance CNS remyelination in inflammatory conditions. In EAE and cuprizone mouse models of MS, prolonging the ISR protects oligodendrocytes and enhances remyelination. The use of a selective estrogen modulator, bazedoxifene (BZA), increases the number of oligodendrocytes in cuprizone mice, and its combination with Sephin1 (an agent that prolongs ISR) enhanced remyelination [129].

ATF4 is an important downstream effector of the ISR; hence, its role has been investigated in the context of EAE development. Unexpectedly, evidence suggests that specific deletion of ATF4 in oligodendrocytes does not exert discernible impact on EAE severity when compared to their controls [130]. Additionally, ATF4 deficiency in oligodendrocytes does not significantly alter their number, nor changes axon degeneration in EAE lesions. These findings suggest while the PERK-eIF2α pathway protects oligodendrocytes in EAE mice, its downstream effector, ATF4, does not have a significant role in regulating oligodendrocytes’ survival and axonal degeneration during EAE [130]. Recently, stress granules have been shown to exert neuroprotective effects in MS by sequestering pro-apoptotic factors, hence protecting mRNA and proteins from degradation. This was shown in the corpus callosum of cuprizone-induced demyelinating mouse model in which Sephin1 treatment allowed differentiation of oligodendrocyte progenitor cells (OPCs) into oligodendrocytes in the context of inflammatory stress [131]. This study showed the presence of astrocytic IFNγ in GFAP-tTA; TRE-IFN-γ transgenic mice underpins Sephin 1’s effects [131]. Interestingly, Sephin1 extended the IFNγ-triggered ISR by enhancing the levels of p-eIF2α that resulted in reduced protein synthesis and the formation of RNA stress granules in oligodendrocytes [131]. Studies in a 9-week-old EAE mouse model, which recapitulates the inflammatory aspects of MS, have shown suppressing the ISR with 2BAct results in a partial loss of the protective effects of Sephin1 suggesting that Sephin1 may protect the CNS from inflammation by enhancing the ISR. These findings have provided initial evidence that Sephin1 may protect oligodendrocytes through the formation of RNA stress granules [131]. Although these EAE findings aid in understanding the role of the ISR in MS, these studies have their limitations. MS is a disease that progresses over time, and its severity can increase with age. Thus, EAE findings fail to address the relevance of the ISR in progressive MS. Further investigation in chronic progressive models of MS would allow uncovering the role of ISR in disease progression and aging in MS. Future research will also need to focus on understanding how ISR modulate activity of mature and older oligodendrocytes, and its role in remyelination.

Recent studies from active and inactive human MS lesions have also shown the presence of stress granules in oligodendrocytes, which has been also confirmed in cultures of human oligodendrocytes under metabolic stress [132]. These studies revealed that stress granules persist in oligodendrocytes while pro-inflammatory cytokines are present in culture media [132]. Interestingly, while PERK activity is shown to be important for oligodendrocytes under injury, neither PERK activation nor deletion alters the viability or myelinating capacity of oligodendrocytes under homeostasis [127, 133]. Similarly, the conditional deletion of ATF4 in oligodendrocytes has shown negligible effects in the CNS under homeostasis; with little effect on myelination, and a similar number of oligodendrocytes and axons between the ATF4 conditional knockout and the wild-type mice [130]. Altogether, emerging evidence suggests the involvement of ISR pathways in regulating oligodendrocytes in MS; however, further research is required to elucidate the underlying mechanisms by which ISR contributes to MS pathogenesis and progression. Future research is required to elucidate the underlying mechanisms by which mitochondrial dysfunction (a known mechanism of MS pathogenesis) [134–136] engages in a crosstalk with ISR during MS progression and its pathogenesis.

### The role of ISR in Alzheimer’s Disease (AD)

AD is a neurodegenerative condition commonly characterized by memory loss, depression, disorientation, behavioral changes, and motor-related impairments [137]. AD causes synaptic and neuronal loss, oxidative damage, neuroinflammation, among others. Plaques of amyloid-beta (Aβ) and neurofibrillary tangles (NFTs), which are phosphorylated aggregates of tau protein, are hallmarks of AD. As reviewed previously, Aβ can disturb the function of proteasomes and lysosomes, impair calcium homeostasis, and enhance the formation of NFTs in neurons [138]. Furthermore, the continuous accumulation of Aβ and phosphorylated tau can result in abnormal protein folding and subsequent ER stress [138]. Early studies on post-mortem AD brain showed upregulation of p-PERK and p-eIF2α in the hippocampus and frontal cortex. While the results of this study were initially associated with the UPR only, it is important to consider that the ISR and the UPR converge in the phosphorylation of PERK and eIF2α, which may suggest the involvement of both pathways [139]. Subsequent studies established a relationship between tau phosphorylation and ER stress. Higher expression of p-PERK and p-eIF2α were reported in the hippocampus of P301L aged mice, a transgenic mouse model that develops tau pathologies [140]. Additionally, primary cortical neurons from embryonic day 17 Sprague–Dawley rats, treated with the ER-stressor thapsigargin showed tau hyperphosphorylation and cleavage. Interestingly, inducing tau hyperphosphorylation in vitro with okadaic acid led to increased immunoreactivity of p-PERK accompanied by a significant increase in p-eIF2α [140]. Consistent with these findings, studies in 6-month-old P301L mice showed increased expression of p-eIF2α and ATF4, and low protein synthesis compared to wild-type mice [141]. Interestingly, the levels of spliced XBP1 and ATF6 did not increase at the 6-month mark, which rules out the involvement of the UPR and suggests the presence of ISR signaling. Additionally, it was found that activation of PERK-eIF2α contributed to the pathological phosphorylation of tau in rTg4510 mice, a mouse model of tau pathology with pronounced neurodegeneration similar to human tauopathies. Contribution of PERK-eIF2α axis to tau pathology was further supported when suppressing PERK activity in P301L mice with the PERK-specific inhibitor GSK2606414 restored the rate of protein synthesis by downregulating p-PERK, p-eIF2α, and ATF4. Furthermore, P301L mice treated with GSK2606414 show a significantly higher number of CA1 pyramidal neurons and ameliorate brain atrophy suggesting PERK inhibition may provide neuroprotection [141].

Other studies suggest that PERK-eIF2α signaling might be beneficial in AD [142]. Knockdown of PERK in SK-N-SH human neuroblastoma cell lines enhances Aβ-mediated cell death in neurons by suppressing eIF2α and the Grp78/Bip ER chaperon [142]. Promoting PERK signaling is also shown to mitigate tau pathologies both in vivo and in vitro [143]*.* In an in vitro model of tauopathy in human neurons, enhancing PERK signaling through pharmacological and genetic approaches reduces tau phosphorylation and tau conformational changes that promote neuronal survival. Moreover, PERK activation significantly improves memory and locomotion in P301S tau mice by reducing tau pathology, promoting dendritic spine density and attenuating motoneuron loss [143]. Human studies have identified the presence of EIF2AK3 variants (encoding for PERK) in Dutch patients with AD, indicating that these variants may result in an increased risk of developing the disease [144]. Follow-up studies have further demonstrated that PERK has a role in tau protein aggregation. In vitro assessments indicate that inhibiting PERK directly (hence decreasing eIF2α phosphorylation) promotes tau aggregation and increasing eIF2α phosphorylation prevents tau aggregation in Biosensor cells. In accordance with these findings, post-mortem brain samples from AD donors show that PERK signaling is downregulated in the hippocampus [145].

Other EIF2A kinases have been implicated in AD. Early studies showed that Aβ peptides promote PKR phosphorylation in rat primary neurons and suggested that increased intracellular calcium is important for the Aβ peptide-activation of the PKR-eIF2α pathway [146]. Recently it has been proposed that PKR may directly regulate tau expression in AD as in vitro assessments on OLN-T40AS cells showed that PKR directly phosphorylates several abnormal residues within tau [147]. Furthermore, changes in PKR expression led to corresponding changes tau mRNA and protein levels in OLN-T40AS cells. Brain slices from the AD rTg4510 mouse model treated with a PKR inhibitor show reduced phosphorylation of soluble tau [147]. GCN2 has also been investigated; however, evidence shows that this kinase does not have a significant role in AD. Findings from 5XFAD mice (a mouse model of severe amyloid pathology) with GCN2 deletion suggest that overactivation of PERK facilitates phosphorylation of eIF2α, and aggravates the expression of hippocampal BACE1 and ATF4, failing to rescue memory deficits in a fear conditioning test [116].

In AD, an imbalance in mitochondrial proteastasis due to accumulation of unfolded proteins in the mitochondrial matrix is shown to increase the expression of key genes that are involved in stabilization of mitochondria during Aβ-mediated proteotoxicity [148]. In one such study, Beck and colleagues showed that UPR^mt^ is prominently activated in frontal cortex of both sporadic and familial AD human postmortem tissue samples [149]. Another study by Shen and colleagues in APPsw/PS1dE9 transgenic mice and SH-SY5Y cell line revealed that an increase in the expression of UPR^mt^ proteins such as Hsp60, CLPP and Htr/Omi is observed in 3-month-old mice while remained unchanged in 9-month-old mice comparing their age-matched wildtype-control [65]. This study suggests that activation of UPR^mt^ decreases with aging during the process of mitochondrial dysfunction in AD [65]. The authors also found that sphingolipid biosynthesis and mevalonate pathways are necessary for the activation of UPR^mt^ induced by Aβ, and inhibiting these pathways in SH-SY5Y cells prevents UPR^mt^ activation, aggravates abnormal mitochondrial structure, increases ROS levels, and exacerbates cytotoxicity mediated by Aβ plaques. These changes are known to have modulatory effects on ISR, limiting the beneficial effects of UPR^mt^. Taken together, growing evidence from preclinical studies have demonstrated a link between the ISR and AD pathology.

Preclinical AD models have been crucial to elucidate the role of ISR in AD and to assess how its inhibition with ISRIB would impact AD pathophysiology. Current evidence from mouse models suggests a controversial role for ISR in AD. For instance, there are animal studies that show contrasting effects of ATF-4 in mediating Aβ plaque induced neuropathology. An intra-axonal translation of ATF-4 was reported to induce Aβ_1-42_ mediated neurodegeneration in a retrograde manner [113, 150]. Another work also showed that ISRIB treatment prevents Aβ-induced cell death in the neuronal cell line PC12 [151]. These studies proposed that this effect is likely through the inhibition of ATF4 with no impact on Aβ production [151]. In contrast, recent studies have identified that the small molecule ISRIB provides neuroprotection against the disruptive effects of Aβ on synaptic integrity and cognition in a rat model of sporadic AD [152]. It was suggested that this effect may be due to a restoration of Aβ-induced aberrant protein synthesis and increased expression of ATF4 in the hippocampus [152]. There are other reports that also demonstrate the beneficial effects of PERK and eIF2α phosphorylation in tau pathology [142, 143]. Overall, these examples suggest a controversial role of ISR in AD pathology that requires further investigation.

To critically interpret these conflicting reports, it is crucial to consider that these studies have their limitations. Firstly, current AD models do not fully replicate the complex pathophysiology of this disease. As an age-related neurodegenerative disease, it is important to consider that most animal models have short lifespans, which limits the ability to effectively mimic disease progression. Furthermore, findings related to the protective effects of ISR activation observed in these models could be a secondary effect to the artificial overexpression of proteins. Understanding the dynamic interplay between disease progression and its relationship with different ISR kinases, and cellular stress responses are among the critical points to address. Additionally, the timing and duration of the ISR can impact tau pathology and AD progression. Overall, due to the complexity of AD, further investigation on the effects of pharmacological modulation on the ISR and its different branches at different stages would help elucidate the mechanisms of this pathology and help identify potential therapeutic approaches.

### The ISR and Parkinson’s Disease (PD)

PD is another neurodegenerative disease that impacts dopaminergic neurons in the substantia nigra causing disruption in nigrostriatal dopaminergic innervation in the brain. PD is characterized by the misfolding aggregation of α-synuclein that contributes to the formation of Lewy bodies, a hallmark of this disease [153].The formation of these aggregates, along with an impaired protein clearance by the ubiquitin–proteasome and autophagy-lysosomal systems, mitochondrial dysfunction, neuroinflammation, and oxidative stress collectively contribute to neurodegeneration [154]. Another key aspect of this disease is the failure of mitochondrial quality control, which plays a major role in neuronal survival. Mitophagy, the process where excessive or damaged mitochondria are degraded by lysosomal hydrolases, is crucial for maintaining homeostasis [155]. Recent findings demonstrated that the HRI branch of the ISR selectively induces mitophagy via the mitochondrial localization of peIF2α [156]. In this study, K562 engineered cells, which express the mitophagy reporter mito-mKeima, were treated with either the BTdCPU or Salubrinal. Flow cytometry identified an increase in HRI-dependent mitophagy, and both treatments were associated with the accumulation of ATF4 and p-eIF2α protein levels [156]. Additionally, it was reported that the HRI mitophagy pathway is activated in parallel with the mitophagy pathway regulated by the PD-associated genes PINK1 and PARKIN, with distinct underlying mechanisms. Therefore, it is suggested that the HRI pathway, which normally results in translational initiation, can trigger mitophagy in response to mitochondrial damage [156]. Although these are promising findings that could be associated with a mechanism of PD, it should be noted that the study was limited to K562 and HeLa cell lines, which are not relevant in vitro PD models. Further studies with PD cell lines will be required to confirm an association between HRI-dependent mitophagy and neuronal integrity.

PD is part of a group of diseases known for abnormal proteostasis and accumulation of misfolded proteins that result in neuronal dysfunction [157]. ER stress and possibly ISR have been associated with PD. Early studies on post-mortem brain samples identified the presence of p-PERK and p-eIF2α in the substantia nigra of PD brain in which 5–20% of the α-synuclein positive neurons were immunoreactive for p-PERK while it was absent in the control non-PD brain [158]. Moreover, over-expression of truncated α-synuclein in HEK 293 cell line, which does not express wild-type α-synuclein, results in high expression of GRP78/BiP. Interestingly, under glucose deprivation, dopaminergic neurons differentiated from SH-SY5Y cells have shown formation of intracellular aggregates of α-synuclein, and some of these aggregates expressed GRP78/BiP and ATF4 [159]. In agreement with these findings, dopaminergic neurons of the substantia nigra in SYN120 transgenic mice (a model of PD that overexpresses α-synuclein) express higher levels of GRP78/BiP as compared to their wildtype controls [15, 159]. Altogether, in vitro and in vivo evidence from PD models suggest the involvement of PERK signaling of the ISR in the disease.

Studies by Bouman and colleagues reported that ATF4 is involved in the transcription of Parkin, a pro-survival protein that acts as a regulator for protein breakdown and mitochondrial integrity [160]. In this study, SH-SY5Y cells and primary mouse cortical neurons with mitochondrial damage showed increased transcription of Parkin and BiP mRNA. Furthermore, Parkin is upregulated in response to thapsigargin- and tunicamycin-induced ER stress and is identified as a downstream target of the PERK/ATF4 pathway [160]. Interestingly, ATF4 knockdown in vitro can significantly reduce expression of Parkin. This study determined that Parkin expression upon PERK activation seems to be neuroprotective due to its role in reducing mitochondrial damage [160]. In support of this evidence, another study has identified that ATF4 has a cytoprotective role in PD [161]. Silencing ATF4 promotes cell death of neuronal PC12 cells in the presence of dopaminergic neurotoxins 6-OHDA and MPP + [161]. Conversely, ATF4 overexpression reduces cell death by maintaining the expression of Parkin [161]. Although these studies showed a protective role of PERK/ATF4, a follow-up study from the same group reported that CHOP and ATF4 mediate the transcription of Trib3 (tribbles pseudo kinase 3), a protein with pro-apoptotic function that is highly activated in PC12 cells under 6-OHDA in an in vitro model of PD [162]. These in vitro results were confirmed in human post-mortem PD brain where Trib3 was highly expressed in nigral dopaminergic neurons [162]. Furthermore, sustained over-expression of ATF4 by rAAV in a rat model of PD-like neurodegeneration, induced by human wild type α-synuclein, is shown to promote neuronal cell death in the substantia nigra pars compacta (SNpc) [163]. Studies in a mouse of model PD-like progression reported that ATF4 binds to the E3 ligase parkin promoter thereby regulating its expression in response to mitochondrial and ER stress [164]. These findings show that the increased expression of parkin due to ATF4 is beneficial during mitochondrial damage and ER stress as PD is developed. The brain biopsy tissues from PD patients also exhibit elevated levels of ATF-4 immunostaining in the neuromelanin-positive neurons suggesting that ATF-4 expression is affected by the mean duration of the PD; the longer the progression the higher the ATF-4 expression levels [160].

The synucleinopathy is also known to disrupt the mitochondrial health through dysfunctional membrane potential, degeneration of mitochondrial complex I, disruption of Ca^2+^ homeostasis, and an increased release of cytochrome c [165]. Moreover, an imbalance in mitochondrial proteostasis due to soluble oligomers of prefibrillar α-synuclein is commonly observed in PD [166]. Other known factors that disturb mitochondrial homeostasis in PD include disruption of retromer complex which results in mitochondrial fragmentation due to mutation in VPS35 gene [167]; deficiency of mitochondrial protein kinase; and PTEN-induced putative (PINK)1 which is associated with an autosomal recessive variant (PARK6) of PD [168].

Therapeutically, in efforts to target PERK-ATF4 axis in PD, oral administration of the small molecule GSK2606414 to mice models of PD has resulted in the inhibition of PERK activity in the SNpc after experimental ER stress stimulation with tunicamycin [157]. The inhibitor attenuates 6-OHDA mediated cell death of nigral-dopaminergic neurons in PD mice, which improves motor performance and recovery of dopamine and two synaptic proteins, VAMP (Vesicle-associated membrane protein 2) and SNAP25 (Synaptosomal-Associated Protein, 25 kDa) [157]. A recent study in mouse mesencephalic and cortical neuronal cultures has demonstrated that treatment with PD-relevant neurotoxins, MPP + and 6-OHDA, as well as α-synuclein aggregation results in the sustained expression of ATF4 in the nucleus of dopaminergic neurons [169]. These neurotoxins also promote the expression of proapoptotic factors CHOP, Trib3, and Puma through ATF4 activity in dopaminergic neurons. Interestingly, pharmacological inhibition of PKR in cortical and dopaminergic neurons with imidazole-oxindole or C16 suppressed ATF4 [169]. Furthermore, ATF4 inhibition did not reduce the PD neurotoxin-induced eIF2α phosphorylation that suggests ATF4 regulation is independent of PKR, signifying the role of the PERK-ATF4 signaling in PD [169].

In summary, emerging evidence has identified the ISR as a potential therapeutic target for PD. However, further research is warranted to decipher ISR mechanisms in PD, particularly the controversial findings on the involvement of PERK-ATF4 pathway in both neurodegeneration and neuroprotection processes in PD. Like AD, preclinical models of PD rely on genetic and/or pharmacological manipulation to mimic this pathophysiology in rodents. Hence, it may be argued that the effects of the ISR on neurons could be an artificial response to these stimuli. Given that these current experimental models may not fully replicate the mechanisms of ISR in PD pathology, clinical studies are necessary to provide more insight into the positive or negative effects of the ISR.

### The ISR in Amyotrophic Lateral Sclerosis (ALS)

ALS is a devastating neurodegenerative disease that results in the loss of motor neurons in the CNS and is associated with axonal retraction and neuromuscular denervation. ALS has pathological heterogeneity as it can be caused by mutations in SOD1 (Cu–Zn superoxide dismutase) and FUS (fused in sarcoma) genes, which result in the formation of protein aggregates with different compositions [170]. The common signature of ALS is the aggregation of TDP-43 (TAR DNA-binding protein 43) [171]. TDP-43 is a nuclear RNA/DNA binding protein encoded by the TARDBP gene with important roles in transcriptional regulation, alternative splicing, and mRNA stabilization [171, 172]. With motor neurons injury, TDP-43 is released from the nucleus and its mis-localization aids in forming intracellular aggregates in the cytoplasm that is a hallmark of ALS pathology [171]. Compared to other neurodegenerative disorders, the ISR has been well described in ALS. Early studies on human ALS samples identified upregulation of ISR markers, such as p-eIF2α, in spinal cord neurons [173]. Development of different animal models of ALS has also aided in understanding the role of ISR signaling in regulating motor neurons in ALS. The mutant SOD1 mouse model that mimics familial ALS is known to cause protein misfolding and aggregates formation, thus it is commonly used to study the role and mechanisms of the ISR in ALS complementing human ALS studies. Evidence also suggests high expression of CHOP in neurons, oligodendrocytes, microglia, and astrocytes in the Rexed laminae IX anterior horn of the spinal cord of sporadic ALS patients and the SOD1^G93A^ mouse [173]. Overall, emerging animal studies have provided supportive evidence that ER stress, UPR and the ISR have an important role in ALS pathophysiology.

Given the diversity of genetic mutations that can cause ALS, it is proposed that the ISR can be triggered through different mechanisms. Studies in SOD1 mutants have suggested accumulation of different SOD1 mutant proteins can induce UPR by phosphorylation of IRE1 and PERK [174]. Furthermore, the interaction between the protein Derlin-1 and SOD1 mutants has been proposed as an underlying mechanism for inducing ER stress and UPR activation [174]. Higher levels of ATF6 and p-eIF2α in the motor neurons of transgenic SOD1^G93A^ mice has further substantiated the involvement of UPR in ALS, although the levels of spliced XBP1 or sXBP1 (a downstream marker of the UPR/IRE1 pathway) are not significantly different [175]. These studies proposed that ATF6 activation may occur earlier in ALS than XBP1 by proteolysis, and the latter is upregulated upon de novo synthesis [175]. Interestingly, a high expression level of UPR-related markers like sXBP1, p-eIF2α, GRP78/BiP, and CHOP are also detected in the skeletal muscle of SOD1^G93A^ mice, suggesting that the UPR pathway may be implicated in the muscle atrophy and weakness observed in ALS by affecting muscle cells instead of direct effects on motor neurons and motor pathways [176].

Studies in SOD1 mouse model have reported different activation profile of UPR system based on the type of transgenic mice. Although motor neurons of transgenic SOD1^G93A^ mice have shown increased UPR activity via PERK and ATF6, transgenic SOD1^G85R^ mouse model only showed significant activation of PERK signaling in motor neurons through upregulation of ATF4 and CHOP with no changes in the levels of BiP and sXBP1 [177]. Given the heterogeneity of ALS, it is plausible that some mutations result in UPR activity while others activate the ISR, without involving the UPR. A genome-wide screening for activators and suppressors of the ALS-associated gene C9ORF72A using the CRISPR Cas9 system supports this hypothesis by identifying a robust activity of the ISR, but not the UPR [178]. More recent findings demonstrated that patients with C9ORF72 repeat expansion mutations show increased levels of phosphorylated PKR, suggesting that other ISR-related eIF2α kinases are involved in ALS [179]. Further studies from a Drosophila model and in rat primary neurons have also provided evidence that the PERK inhibitor GSK2606414 reduces TDP43 toxicity, supporting a role for the ISR in neurotoxicity [180]. SOD1^G85R^/GADD34^+/ΔC^ mice with a mutation in GADD34 show an enhanced phosphorylation of eIF2α in spinal cord homogenates, indicating prolonged ISR [181]. Additionally, SOD1^G85R^/GADD34^+/ΔC^ mice show a delayed onset of the disease with higher survival rates when compared to the SOD1^G85R^ control. Thus, it has been proposed that the beneficial effects observed in the SOD1^G85R^/GADD34^+/ΔC^ are attributed to an enhanced ISR that delays accumulation and formation of mutant SOD1 aggregates.

Evidence from three different familial ALS mouse models has helped classify two types of motor neurons according to their vulnerability or resistance to ER stress [182]. Interestingly, fast-fatigable motor neurons are more vulnerable and prone to developing ER stress and show an increased expression of p-eIF2α, while the fatigue-resistant neurons demonstrate a delayed response to ER stress [182]. This work provided a mechanism underpinning early disease manifestations related to the susceptibility of motor neurons, and late manifestations in the resistant neurons [182], and it might indicate a relationship between the ISR and highly active motor neurons with high metabolic demands. Recent work has provided more insight into the role of the ISR by using the rNLS8 mouse model, which mimics aggregates of insoluble cytoplasmic TDP-43 observed in ALS [183]. Transcriptomic and protein analyses of rNLS8 mice indicate cortical upregulation of ISR- and apoptosis-related genes ATF4, CHOP, Bid, Gadd45, Trp53 before the disease onset. Upregulation of CHOP at the onset of the disease and increased levels of cleaved caspase-3 in the cortical neurons of rNLS8 mice suggested a role for CHOP in apoptosis-mediated neurodegeneration in ALS. However, further evidence showed that the knockdown of CHOP in rNLS8 mice did not alter the TDP-43 pathology and caspase-3 activation, suggesting other pathways that may account for activation [183]. Intriguingly, a recent study has suggested that the ISR might have a protective rather than pro-apoptotic role in ALS [184]. This in vitro study on human P525L FUS neurons indicate that FUS mutations result in the activation of the heat-shock response and the ISR [184]. However, cell survival was similar in mutant and wild-type neurons under various stress conditions. This suggests that over-activation of stress response pathways in mutant cells may protect them from susceptibility to cell death. However, when the ISR was acutely inhibited under stress conditions the outcome did not change, suggesting that the ISR on its own may not be responsible for these protective effects. Altogether, while further studies are required to clarify these findings, it is plausible that the ISR may act as an early protective mechanism in response to the accumulation of FUS in the cytoplasm by preventing cell death, however, it can turn into a toxic mechanism over time [184].

The ER-mitochondrial crosstalk is also known to regulate synaptic transmission, and its impairment can lead to dysfunctional synapses which is observed in many neurodegenerative disorders like ALS [185]. Accumulating evidence also indicates that mitochondrial dysfunction plays an important role in ALS pathogenesis, as ER-mitochondrial physical and functional connection contributes to the calcium homeostasis and lipids biosynthesis [186]. These reports have indicated a pivotal role for SOD1 mediated mitochondrial dysfunction. Recent evidence in familial ALS cases has also unraveled that mutations like TARDBP and C9ORF72 contribute to morphological defects in mitochondria [187]. The role of mitochondria and ER crosstalk in modulation of ISR during ALS pathogenesis requires further investigations.

Preclinical research has been conducted to develop therapeutic strategies for modulating the ISR in ALS. Guanabenz, a non-selective inhibitor of ER stress, has been tested in ALS. In animal models of ALS, Guanabenz, that is commonly used to treat hypertension, has been identified to induce eIF2α dephosphorylation and persistent activity of the ISR, allowing the clearance of misfolded proteins and prolonging motor neurons survival [188]. A multicentre, randomized, double-blind phase 2 clinical trial has reported that guanabenz at two doses, 64 and 32 mg, can slow the progression of ALS in patients with bulbar onset, which is the most homogenous phenotype of ALS [189]. DNL343 is another candidate treatment that has been investigated in A recent phase I trial in patients with ALS for its potential as eIF2B agonist. DNL343 is shown to attenuate the formation of ISR stress granules in vitro, [190]. The results of the phase I trial have identified the safety of DNL343 in humans; however, its efficacy is yet to be tested in future phase II trials.

## Conclusions and future perspectives

The integrated stress response has gained increasing attention in the past two decades for its role in pathogenesis and progression of neurodegenerative diseases. There is now more understanding of how the eIF2α kinases detect various types of stress and converge on the phosphorylation of eIF2α to regulate the CNS [116]. It is also increasingly acknowledged that the ISR is a critical pathway that ensures cells can respond to stress, maintain proteostasis, and adapt to changing conditions. The ISR also plays pivotal role in maintaining CNS homeostasis, particularly during neurodevelopment and memory consolidation, where various eIF2α kinases and downstream signaling pathways collectively contribute to the intricate regulation of synaptic plasticity in the brain [45].

Given the important role of ISR in regulating proteostasis, learning, and memory formation, there has been growing interest in identifying mechanisms that link the ISR and neurodegenerative disorders, where proteotoxicity and cognition decline are involved. Recent animal and human studies have confirmed the involvement of ISR in pathogenesis of various neurodegenerative disorders. Hence, efforts have been made to develop therapeutic strategies to target ISR pathways. In MS preclinical studies, where demyelination and oligodendrocyte loss are the hallmarks of the disease, current evidence suggests that prolonging the ISR with Sephin1 might be a potential strategy to promote remyelination [128, 129, 131]. In AD, pharmacologic and genetic enhancement of PERK signaling has shown promise to reduce tau phosphorylation [143]. Interestingly, PKR enhancement results in tau phosphorylation, while its inhibition has the opposite effect [147]. These findings suggest the complexity of ISR signaling and the differential response of various kinases in the pathway, signifying the importance of this information for developing therapeutic approaches. In PD, the PERK-ATF4 pathway has been identified as a potential target since inhibiting PERK activity with GSK2606414 has shown neuroprotective effects and preserves dopaminergic neurons in PD animal models [157]. The ISR has been studied more extensively in ALS and Guanabenz is currently being evaluated as a therapeutic strategy that has shown encouraging results in a phase II clinical trial [189].

In conclusion, ISR has emerged as a promising target in neurodegenerative diseases. Our current understanding of cellular communication of stress responses to mount a cytoprotective role (at single cell level) or to mitigate the overall stress in a collective cellular context is a subject of further investigation. Thus, it is essential to develop insights into the outcomes of targeting the stress response pathways by modulating the ISR in different cell types and disease phenotypes. This knowledge is key to uncovering the regulatory mechanisms of different stress responses across the organism and would facilitate development of effective pharmacological based treatments. To this end, the timing of ISR activation during the development of neurodegenerative phenotypes and linking these events with other cellular pathways such as mitophagy, oxidative stress, ER stress, immunomodulatory response, and metabolism are among the most crucial pathways to unravel. Another major knowledge gap in this area is how the pro-survival and pro-apoptotic pathways are mediated by the eIF2α kinases or the underlying mechanisms that direct the preferential translation of certain mRNAs. Altogether, further understanding of the dual roles that ISR play in survival and apoptosis is crucial for developing effective therapeutic interventions to modulate the ISR in these neurodegenerative conditions.
